# The promise of cardiomyocyte computational models for drug safety

**DOI:** 10.1186/s40779-025-00675-3

**Published:** 2025-12-05

**Authors:** Lei Wang, Blanca Rodriguez

**Affiliations:** 1 Beijing National Center for Applied Mathematics, Capital Normal University, Beijing, 100048 China; 2 Department of Computer Science, University of Oxford, Oxford, OX1 3QD UK

**Keywords:** Cardiomyocyte computational models, Drug safety testing, Cardiac electrophysiology, In silico clinical trials, Artificial intelligence (AI)

Dear Editor,

Drug development stands at a crossroads. Traditional animal testing for cardiac safety faces growing scientific and ethical challenges, while computational alternatives are demonstrating valuable predictive capabilities. Cardiomyocyte computational models, rooted in concepts developed over 6 decades ago, now represent a mature and viable approach for pharmaceutical research, particularly when integrated with modern experimental platforms. This letter reviews the application of cardiac electrophysiology models in drug safety evaluation, a field where they offer transformative potential. We focus specifically on cardiac electrical activity relevant to drug safety, leaving the equally important domain of cardiac mechanics for future discussion.

## From foundational equations to mechanistic understanding

The mathematical modeling of cardiomyocytes traces its lineage to the seminal Hodgkin-Huxley equations, adapted for cardiac cells by Noble in 1962. This pioneering work demonstrated that complex physiological behaviors could be captured through mathematical formalisms, giving rise to computational physiology as a legitimate scientific discipline. What began as a theoretical exercise has evolved into a practical tool with real-world applications.

The subsequent evolution of these models exemplifies the power of iterative collaboration between experimentalists and computational scientists. A representative case is the development of the human ventricular ToR-ORd model [1], a next-generation human ventricular myocyte model that builds upon the widely used ORd framework. The ToR-ORd model was created to resolve key inconsistencies between the ORd model and experimental human data, such as an excessively high action-potential plateau, unrealistic calcium-transient kinetics, and incorrect inotropic responses to sodium-channel blockade. To address these issues, the ToR-ORd model reformulated critical ionic currents, most notably the L-type calcium and rapid delayed-rectifier potassium currents, using biophysically consistent equations and human-specific datasets. Through a rigorously separated process of model calibration and independent validation, the ToR-ORd model reproduced human ventricular electrophysiology and calcium-handling dynamics with markedly improved quantitative accuracy across healthy, diseased, and drug-block conditions. It accurately predicted experimentally observed phenomena such as the negative inotropic effect of sodium blockers, early after-depolarizations under human ether-a-go-go-related gene (hERG) block, and physiological electrocardiogram (ECG) waveforms at the whole-heart level. This example illustrates how sustained experimental-computational feedback not only increases model fidelity but also expands predictive capacity. Modern simulations now incorporate intricate details of ion channels, transporters, and calcium handling dynamics, capturing differences across species, developmental stages, and disease states with remarkable fidelity. Critically, model development requires extensive experimental data to inform parameters and mathematical equations, while model predictions must be validated against experimental observations for each specific application. These models serve multiple purposes: testing mechanistic hypotheses, predicting responses to novel conditions, identifying therapeutic targets, and guiding experimental design, always through close partnership with experimental validation.

## Computational platforms for drug safety assessment

To harness these capabilities, several software platforms have emerged. The Virtual Assay software developed at the University of Oxford [2] represents significant progress in translating cardiomyocyte computational models to practical drug safety assessment. Additionally, open-source platforms such as openCARP and MonoAlg3D, as well as community-driven initiatives like the Virtual Physiological Human Project, provide valuable frameworks for cardiac modeling more broadly, supporting reproducibility and collaborative research across diverse applications beyond drug safety testing.

These computational platforms address fundamental limitations of traditional preclinical testing approaches. Simulations using computational models offer several distinctive advantages over conventional animal studies, especially when combined with human-based in vitro technologies. First, they eliminate species translation barriers by directly incorporating human-specific ion channel kinetics and cellular electrophysiology, avoiding the extrapolation uncertainties inherent in animal-to-human translation. Second, these platforms enable population-scale simulations, running hundreds to thousands of virtual cells with diverse electrophysiological phenotypes to reveal how drug effects distribute across genetically varied populations, a capability beyond the reach of traditional animal experiments. Third, computational approaches provide mechanistic transparency, decomposing the causal chain from drug-channel interactions through cellular responses to tissue-level effects, offering insights into why certain responses occur rather than simply whether they occur. Fourth, these methods achieve substantial improvements in speed and resource efficiency compared with animal studies, enabling rapid iteration during drug development. Finally, computational models align with evolving regulatory frameworks that increasingly recognize alternatives to animal testing, particularly as initiatives like the U.S. Food and Drug Administration (FDA) Modernization Act 2.0 and comprehensive in vitro proarrhythmia assay (CiPA) initiative.

These advantages position computational models as valuable complements to experimental approaches, offering capabilities that traditional methods cannot easily replicate while requiring careful validation against clinical observations.

## From virtual assays to in silico clinical trials

The concept of in silico clinical trials extends computational modeling to population-level predictions [3]. These virtual studies simulate entire patient cohorts with realistic variability in drug responses. Unlike traditional trials that recruit specific populations, computational studies explore diverse scenarios, including rare genetic variants and extreme dosing conditions. This permits ethical exploration of dangerous circumstances such as severe overdoses or multi-drug interactions that would be unacceptable in human trials. Such capabilities optimize trial designs before recruiting a single patient.

Regulatory landscapes increasingly recognize computational evidence as they prioritise human-based technologies. As a precursor of the FDA’s modernization act 2.0, the FDA’s CiPA initiative formally incorporated human-based computational modeling and in vitro methods into cardiac safety assessment frameworks [4]. This represents a significant shift in how regulatory agencies evaluate drug safety. The pharmaceutical industry has responded accordingly, with computer modeling and simulation forming an integral part of drug discovery and development processes [5], illustrating how in silico methods are increasingly embedded within pharmaceutical workflows. Other major companies, such as Roche and Novartis, have also established dedicated computational modeling divisions that integrate mechanistic simulations into preclinical safety evaluation and compound optimization. Beyond traditional pharmaceutical organizations, emerging technology enterprises such as HeartFlow, InsilicoTrials*,* ELEM Biotech, or Numericor are demonstrating how large-scale computational modeling and data-driven methodologies can markedly accelerate the development of medical therapies, refine or replace animal testing, and enhance the predictive assessment of medical product safety. The FDA now accepts modeling data as supporting evidence for drug approvals when combined with traditional experimental validation [6]. These collective efforts underscore a growing recognition that in silico modeling is not merely a supportive tool, but a strategic driver of innovation and efficiency across the drug development pipeline.

## The artificial intelligence (AI) frontier: from machine learning to autonomous model construction

The convergence of AI with computational physiology promises to accelerate progress further. Machine learning algorithms identify complex patterns in large datasets, revealing drug-safety relationships invisible to conventional analysis. For instance, deep learning approaches have shown promise in predicting drug-induced arrhythmia directly from action potential recordings of human induced pluripotent stem cell (iPSC)-derived cardiomyocytes, achieving accurate risk stratification while discerning how patient genetics influence drug response [7]. Another study used Gaussian process classification integrated with whole-heart simulations to identify the boundary between safe and arrhythmic drug concentrations, correctly classifying 22 common drugs based on their effects on rapid delayed rectifier potassium and L-type calcium currents [8]. Beyond prediction, large language models are beginning to assist in literature synthesis, hypothesis generation, and parameter optimization, streamlining the model development process.

Most remarkably, systems like CellForge demonstrate how AI agents can autonomously construct and optimize computational cell models directly from experimental data and research objectives [9]. This represents a future where model creation becomes automated, vastly accelerating our ability to generate predictive digital twins of human cells.

## Toward personalized and mechanistic drug development

Cardiomyocyte computational models represent a fundamental shift toward a mechanistic understanding of drug action on cardiac electrophysiology. However, their predictive power depends critically on high-quality experimental data. The development and validation of models for diverse populations, including pediatric patients and individuals with genetic cardiomyopathies, requires experimental platforms that can provide relevant physiological measurements. Engineered heart tissues (EHTs), built from human pluripotent stem cell-derived cardiomyocytes [10], have emerged as particularly valuable tools in this context. Unlike animal models, EHTs can be generated from patient-specific cells carrying disease-relevant genetic variants, providing human-relevant data that directly inform model parameters and enable validation of computational predictions across different genetic backgrounds and disease states.

This experimental-computational partnership creates a powerful cycle of refinement. EHTs platforms generate detailed measurements of ion channel function, action potential morphology, and calcium handling under controlled conditions, data that computational models require for accurate parameterization. Conversely, computational models can predict which experimental measurements would be most informative, guiding efficient use of EHTs resources. Through this iterative process, models gain the mechanistic foundation needed for principled extrapolation across developmental stages and pathological conditions.

The translational potential of this approach is substantial. Models validated against EHTs data from healthy cardiomyocytes and those carrying disease-associated mutations can predict drug responses in patient populations that would be difficult or impossible to study directly. This capability addresses critical gaps in current drug development, where pediatric patients and individuals with rare genetic conditions are often excluded from clinical trials. Looking forward, this integrated framework could enable truly personalized medicine, where a patient’s genetic information and physiological measurements inform model parameters to predict both drug efficacy and safety before treatment begins, transforming how we approach cardiac pharmacotherapy.

## Conclusions

The future of drug development and safety testing lies not in laboratory dishes alone, but in sophisticated integration of computational platforms with cutting-edge experimental methods like EHTs. The evidence for this integrated approach is strong, and regulatory acceptance continues to grow through initiatives like CiPA. Computational models offer distinctive advantages in addressing the limitations of traditional animal testing: providing human-relevant predictions, population-scale insights, mechanistic understanding, and alignment with evolving ethical standards.

We call on researchers, pharmaceutical developers, and regulatory agencies to champion interdisciplinary collaborations that leverage computational cardiomyocyte models alongside experimental validation. The widespread adoption of these integrated computational-experimental approaches is not merely an opportunity but essential for building a safer, more efficient, and more humane drug development pipeline.

## Data Availability

Not applicable.

## Abbreviations

AI: Artificial intelligence
FDA: U.S. Food and Drug Administration
CiPA: Comprehensive in vitro proarrhythmia assay
ECG: Electrocardiogram
EHTs: Engineered heart tissues
hERG: Human ether-a-go-go-related gene

## References

[1] Tomek J, Bueno-Orovio A, Passini E, Zhou X, Minchole A, Britton O, et al. Development, calibration, and validation of a novel human ventricular myocyte model in health, disease, and drug block. Elife. 2019;8:e48890. 31868580 10.7554/eLife.48890 PMC6970534

[2] Passini E, Zhou X, Trovato C, Britton OJ, Bueno-Orovio A, Rodriguez B. The virtual assay software for human in silico drug trials to augment drug cardiac testing. J Comput Sci. 2020;45:101163.

[3] Passini E, Britton OJ, Lu HR, Rohrbacher J, Hermans AN, Gallacher DJ, et al. Human in silico drug trials demonstrate higher accuracy than animal models in predicting clinical pro-arrhythmic cardiotoxicity. Front Physiol. 2017;8:668. 28955244 10.3389/fphys.2017.00668 PMC5601077

[4] Strauss DG, Gintant G, Li Z, Wu W, Blinova K, Vicente J, et al. Comprehensive in vitro proarrhythmia assay (CiPA) update from a cardiac Safety research consortium/health and environmental sciences institute/FDA meeting. Ther Innov Regul Sci. 2019;53:519–25. 30157676 10.1177/2168479018795117

[5] Zhou X, Qu Y, Passini E, Bueno-Orovio A, Liu Y, Vargas HM, et al. Blinded in silico drug trial reveals the minimum set of ion channels for torsades de pointes risk assessment. Front Pharmacol. 2020;10:643. 10.3389/fphar.2019.01643 PMC7003137 32082155

[6] Fang L, Tsakalozou E, Polli JE. Paving the path for increased technological innovation, strategic decision making and regulatory acceptance of modeling and simulation approaches. Pharm Res. 2025;42(5):727–9. 40437346 10.1007/s11095-025-03868-6

[7] Serrano R, Feyen DA, Bruyneel AA, Hnatiuk AP, Vu MM, Amatya PL, et al. A deep learning platform to assess drug proarrhythmia risk. Cell Stem Cell. 2023;30(1):86-95.e4. 36563695 10.1016/j.stem.2022.12.002 PMC9924077

[8] Sahli-Costabal F, Seo K, Ashley E, Kuhl E. Classifying drugs by their arrhythmogenic risk using machine learning. Biophys J. 2020;118(5):1165–76. 32023435 10.1016/j.bpj.2020.01.012 PMC7063479

[9] Tang X, Yu Z, Chen J, Cui Y, Shao D, Wang W, et al. CellForge: agentic design of virtual cell models. arXiv:2508.02276 [Preprint]. 2025

[10] Bremner SB, Gaffney KS, Sniadecki NJ, Mack DL. A change of heart: human cardiac tissue engineering as a platform for drug development. Curr Cardiol Rep. 2022;24(5):473–86. 35247166 10.1007/s11886-022-01668-7 PMC8897733

